# The effect of various inhaled asthma medications on the color stability of paediatric dental restorative materials

**DOI:** 10.1186/s12903-024-04118-8

**Published:** 2024-03-26

**Authors:** Merve Candan, Murat Ünal

**Affiliations:** 1 Department of Paediatric Dentistry, Faculty of Dentistry, Eskişehir Osmangazi University, Eskişehir, Turkey; 2 Department of Paediatric Dentistry, Faculty of Dentistry, Sivas Cumhuriyet University, Sivas, Turkey

**Keywords:** Asthma medication, Color stability, Dental restorative materials, Nebulization, Pediatric dentistry

## Abstract

**Background:**

The purpose of the study is to analyse the effects of different inhaled asthma medications (IAMs) on the color change of dental restorative materials (DRMs).

**Methods:**

In total, 192 samples were taken from six different DRMs: [Filtek Z550 (nanohybrid composite), Fusio Liquid Dentin (Self-adhering flowable composite), Filtek Ultimate (nanofilled flowable composite), Dyract XP (compomer), Fuji II LC (resin-modified glass ionomer), Fuji IX Fast (self-cured-packable glass ionomer), (*n* = 32)]. After the initial color values (CIELab) of DRMs were measured by using a spectrophotometer, each sample was exposed to the same IAMs via nebulizer according to the four different inhaled therapies and measurements were repeated on the 7th & 21st days.

**Results:**

In all IAM groups, DRM with the least amount of ΔE was nanohybrid composite, while the highest ΔE was found in Fuji II LC. Among all experimental groups, only Fuji II LC which was administered the combined medication, exceeded the clinically unacceptable threshold (ΔE = 3.3) on 7th & 21st days.

**Conclusions:**

Consequently, important factors affecting the susceptibility to color stability are the type of IAMs, the administration time-dosage, and the type of DRMs.

## Introduction

Asthma is the most common chronic disease among children worldwide and can affect people of all ages. According to the Global Burden of Disease collaboration, approximately 262 million people worldwide suffer from asthma. The disease is treatable with effective medications and methods, and the majority of patients can achieve excellent asthma control. However, inadequate asthma management continues to be a global concern [1].

Asthma and the necessary therapeutic strategies for asthma control can have a significant impact on the oral health of patients with asthma. It has been reported that children and adolescents with asthma are more likely to develop tooth erosion and biofilms than those without asthma [2]. In order to mitigate the impact of asthma and asthma medications on oral health and dental restorations, treatment and medications can be tailored to the individual patient [3].

Nowadays, the increase in the esthetic expectations of both pediatric and adult patients has led to the improvement of a wide variety of dental restorative materials (DRMs). Thus, materials with different inorganic fillers and organic resin matrix types were created. According to the appropriate indication, composites, compomer and glass ionomer cements (GICs) can be used as examples of materials frequently used in dentistry. Considering that these DRMs are widely used in pediatric asthma patients, it is substantial to evaluate the esthetic properties of DRMs that can be preferred in pediatric patients with chronic asthma.

Discoloration in DRMs occurs intrinsically or extrinsically. Extrinsic discoloration is mostly caused by the surface properties of a restoration or by plaque on the DRMs, while intrinsic discoloration is caused by the chemical reactions of the staining solution diffused into the material [4]. The resistance of the materials to intrinsic discoloration is generally related to the water absorption/solubility or contained type of resin matrix of the material [5].

The discoloration of aesthetic dental restorations has been cited as one of the primary reasons for their failure and replacement [6]. Using the Comission Internationale de L’Eclairage (CIE LAB) system formulas, it is important to identify the differences between color change values (ΔE), standardize color stability evaluations, and facilitate a comparison of results from various studies in the literature [7]. In the scoping review conducted in 2023 to evaluate the color changes of various DRMs, the heterogeneity of the included studies was reported in terms of study design, artificial staining procedure, materials analyzed, sample size, surface treatment, and aging. The majority of the evaluated articles, however, utilized the CIELAB color space and CIELAB color difference formula [8].

As a result, color stability is an important requirement in dentistry, especially in anterior esthetic restorations, affecting the selection of restorative materials. For this reason, it is important to be able to choose dental restorations for patients who have to use asthma medications acutely or chronically without compromising their esthetic ideals by dental professionals. When the literature was reviewed, there was only one article evaluating the efficacy of inhaler salbutamol sulfate (Ventolin Nebules, Glaxo Smith Kline, Boronia, Vic., Australia) on the discoloration of composite and GIC [9]. The lack of literature examining the effectiveness of inhaled asthma medications (IAM) on the color stability of DRMs suggests that further exploration is necessary to expand on the information in this subject matter. According to our knowledge, this is the first study investigating the effects of various IAMs on the discoloration of different DRMs.

The inhaler medications that were assessed in the study are colorless liquids that are administered via nebulizer conversion into aerosols. The null hypothesis of the present study is that the effects of various IAMs on the color change of DRMs will not differ.

## Materials and methods

The present in vitro research was carried out with the approval of the Ethics Committee of Sivas Cumhuriyet University Non-Interventional Clinical Research (Approval number: 2019-02/58), and followed the CRIS guidelines for in-vitro studies as discussed in the 2014 concept note.

In the following research, the effects of four different IAM therapies on the color stability of six different DRMs were tested. The technical profiles of DRMs and IAMs are shown in Tables 1 and 2. The Power and Sample Size (PASS) program was used to calculate the sample size; when α = 0.05, β = 0.10, 1-β = 0.90 were accepted, it was decided that each medication group would consist of 48 materials (each subgroup consisted of 8 materials), and the power of the test was *p* = 0.90785 and the effect size was %7.

**Table 1:** The technical profiles of DRMs

**Material**	**Type**	**Composition**	**Manufacturer**	**Lot number**
**Dyract XP**	Polyacid Modified Glass Ionomer (Compomer)	UDMA, TCB resin, TEGDMA, trimethacrylate resin (TMPTMA), dimethacrylate resin, ethyl-4 (dimethylalumino) benzate, BHT, strontium-alumino-sodium-fluoro-phosphorus-silicate glass, strontium fluoride, silicon dioxide, camphorquinone, UV stabilizer	Dentsply, DeTrey, Konstanz, Germany	1902001180
**Filtek Ultimate**	Nanofilled Flowable Composite Resin	Bis-GMA, procrylate resin TEGDMA, ytterbium trifluoride, silica, zircon / silica nanofiller	3M/ESPE, St. Paul, MN, USA	N990357
**Filtek Z550**	Nanohybrid Composite Resin	Bis-GMA, Bis-EMA, PEGDMA, TEGDMA, UDMA, 20 nm silica particles, 0.1-10μ zirconia / silica particles	3M/ESPE, St. Paul, MN, USA	N917719
**Fuji II LC**	Capsulated Light- Cured Resin-Modified Glass Ionomer Cement	HEMA, TEGDMA, methacrylate, tartaric and polyacrylic acid, fluoro-alumino-silicate glass	GC Corporation, Tokyo, Japan	190204 A
**Fuji IX GP Fast**	Capsulated Self-Cured Packable Glass Ionomer	Alumino-fluoro-silicate glass, polyacrylic acid, distilled water, polybasic carboxylic acid	GC Corporation, Tokyo, Japan	180601 A
**Fusio Liquid Dentin**	Self-Adhering Flowable Composite Resin	UDMA, TEGDMA, HEMA, 4-META, silane treated barium glass, silica	Pentron Clinical, Orange, CA, USA	7069226

*Abbreviations*: *Bis-GMA* bisphenol A-Glycidyl Methacrylate, *PEGDMA* polyethylene glycol dimethacrylate, *Bis-EMA* ethoxylated bisphenol-A dimethacrylate, *TEGDMA* triethylene glycol dimethacrylate, *UDMA* urethane dimethacrylate, *HEMA* 2-hydroxyethyl methacylate, *TCB* tetracarboxylic acid modified dimethacrylate *4-META* 4-methacryloyloxyethy trimellitate anhydride

**Table 2:** The technical profiles and daily doseges of IAMs

**Pharmaceutical trade name**	**Content**	**Administration method**	**Maximum allowable daily doses for 6 years and older children**	**Pharmaceutical company**
**Budecort Steri-NEB 0.25 mg/ml nebul**	Each single dose of 2 ml plastic nebul 0.5 mg (0.25 mg per 1 ml) budesonide, disodium edetate, sodium chloride, polysorbate 80, citric acid, monohydrate, sodium citrate, distilled water	Nebulization	4 nebules (1 mg)	Teva, Israel
**Flixotide Nebules 0.5 mg/2 ml nebul**	Each dose 0.5 mg fluticasone propionate, polysorbate 20, sorbitan monolaurate, monosodium phosphate dihydrate, anhydrous dibasic sodium phosphate, sodium chloride, distilled water	Nebulization	4 nebules (2 mg)	Glaxo Smithkline, UK
**Ventoline 2.5 mg/2.5 ml nebul**	3.0 mg salbutamol sulfate equivalent to 2.5 mg salbutamol in each dose, sodium chloride, sulfuric acid, distilled water	Nebulization	8 nebules (20 mg)	Glaxo Smithkline, UK
**Combined Medication** **Ventoline 2.5 mg/2.5 ml nebules** + **Budecort Steri-NEB 0.25 mg/ml nebul**	3.0 mg salbutamol sulfate equivalent to 2.5 mg salbutamol in each dose, sodium chloride, sulfuric acid, distilled water + Each single dose of 2 ml plastic nebul 0.5 mg (0.25 mg per 1 ml) budesonide, disodium edetate, sodium chloride, polysorbate 80, citric acid, monohydrate, sodium citrate, distilled water	Nebulization	8 nebules (20 mg) Ventoline + 4 nebules (1 mg) Budecort	Glaxo Smithkline, UK Teva, Israel

For the standardization of IAMs dosage, the maximum allowable daily dosages (MADDs) for 6 years and older children which are indicated in the prospectuses of each IAM, were accepted. At the same time, the dosage determined for each IAM was approximately the average adult daily dosage. Because equal doses of different IAMs to be investigated under in-vitro conditions may not be applied clinically. Unfortunately, any determined IAM dosage could be higher or lower than the therapeutic dosage of another IAM.

**Fig. 1:**
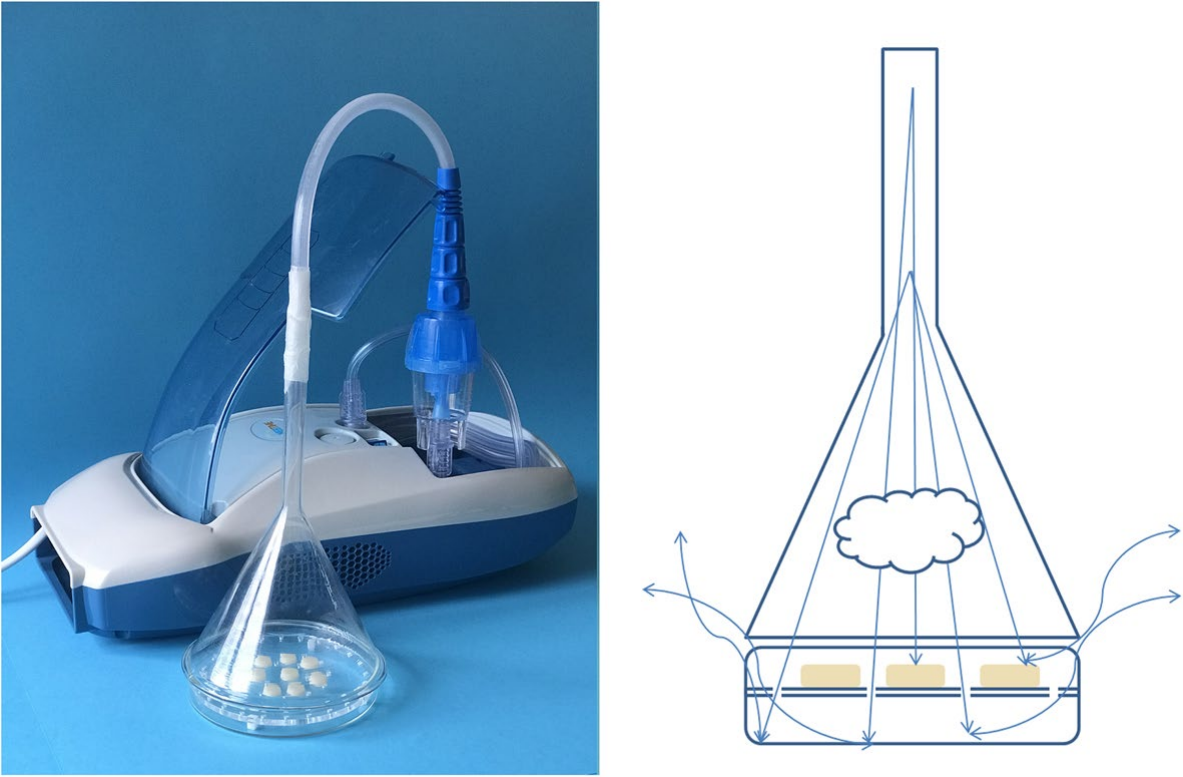
The experimental setup used to administer inhaled asthma medications to the dental restorative materials

The preparation of the samples and administration of IAM followed the methodology used in our previous study [10]. In order to standardize the samples color, the A2 shade was preferred. For the standardization of self-cured-packable and resin modified glass ionomer cements, capsulated DRMs were preferred. 192 sample discs (*n* = 32) were made by using a cylindrical plastic mold (Inner diameter: 8 mm, thickness: 2 mm). After the DRMs were placed in the mold at room temperature (24 °C) according to the manufacturer’s instructions, both sides were covered with a mylar strip. Under the manufacturer’s instructions, resin-based DRMs were polymerized between two flat glasses using an Elipar S10 LED light device with a wavelength range of 430–480 nm and an intensity of 1200 mW/cm2 (3 M ESPE, St. Paul, MN, USA). During polymerization, the light intensity of the LED device was verified using a radiometer (Peng Lim Enterprise Co, LTD, Taiwan) after every 5 specimen. After the samples were extracted from the molds, Sof-Lex discs (3 M ESPE, St. Paul, MN, USA) were used under water for the finishing procedure of the DRMs. A new disc was used for each sample. The finishing procedure of each DRM was performed underwater for 20 s by the same calibrated individual using a low-speed angdurva rotating at approximately 10,000 rpm. After each disc polishing procedure, each sample was thoroughly rinsed for 10 s under running distilled water to remove debris. The IAMs were administered to the DRMs via a nebulizer (Nebtime CNO4, Elmaslar, Turkey) for 21 days. The experimental setup used to administer IAMs to the samples is shown in Fig. 1.

A nebul administration via nebulizer takes 7–8 min on average. According to the prospectus information of the IAMs, half of MADDs for children of the ages of 6 years and older were administered every 12 h. The total dosage of IAM administered each day in this study, is the MADD of each IAMs that can be used in a single day during asthma treatment for aged 6 years and older children. After the IAMs were administered, the samples were washed under distilled water without any physical contact to the IAM administered surfaces and stored in sequentially numbered sterile eppendorf tubes containing freshly prepared artificial saliva solution (ASS) until the next IAM administration. ASSs were changed after each IAM administration. ASS was prepared according to our previous article investigating the surface properties of DRMs [10]. The content of ASS used was; Na_3_PO_4_ (3.90 mM), NaCl (4.29 mM), KCl (17.98 mM), CaCl_2_ (1.10 mM), MgCl_2_ (0.08 mM), H_2_SO_4_ (0.50 mM), NaHCO_3_ (3.27 mM), and distilled water with the pH adjusted to 7.2.

The VITA EasyShade Advance spectrophotometer (VITA Zahnfabrik, Bad Sackingen, Germany) was used to determine the color values of the samples. Color measurements were evaluated based on CIE LAB values. According to the CIE LAB system, the color is defined with L*, a*, and b* values, all of which provide a complete numerical descriptor of the color in a rectangular coordinate system. Before each measurement, the calibration of the device was provided by the white ceramic block on the device. The standard background was used to ensure the standardization of the measurements. During the measurements, the optical tip of the spectrophotometer was positioned parallel to the ground and perpendicular to the samples. All measurements were made in the northern corner of the same room, with the same calibrated person at the same time of day. Three different measurements were made from each sample, and the average of three different measurement values obtained in the CIE LAB system was recorded as the average color value of each material. Color measurements were evaluated initially on the 7th and 21st days. ΔE is a standard measurement that quantifies the difference between two colors that appear on the samples. The color difference between initial − 7th day (ΔE1) and initial – 21st day (ΔE2) was determined according to the formula below:

$$\Delta\mathrm{E}\ast=\lbrack{(\Delta\mathrm{L})}^2+{(\Delta\mathrm{a})}^2+{(\Delta\mathrm{b})}{^2\rbrack}^{1/2}$$

$$\Delta\mathrm{E}\ast={\lbrack({\mathrm L}_1-{{\mathrm L}_0)}^2+({\mathrm a}_1-{{\mathrm a}_0)}^2+({\mathrm b}_1{-{\mathrm b}_0)}^2\rbrack}^{1/2}$$

In the formula, L_0_, a_0_, b_0_ symbolize the initial measurement values, L_1_, a_1_, b_1_ symbolize the second measurement values.

In addition, the National Bureau of Standards ratings (NBS units), used in previous studies [9, 11, 12], to clinically correlate ΔE and NBS units are calculated according to the formula below:

$$\mathrm{NBS}\;\mathrm{units}=\Delta\mathrm{E}\;\times\;0.92$$

NBS units are listed as follows [11, 12]: Trace (0–0.5): Extremely slight change, Slight (0.5–1.5): Slight change, Noticeable (1.5–3): Perceivable change, Appreciable (3–6): Marked change, Much (6–12): Extremely marked change and Very much (≥ 12): Change to another color.

Using the Smile Lite MDP2 polarizing filter (Smile Line, Imier, Switzerland), representative photographs of marked change-detected materials were taken on the initial, 7th, and 21st days. In addition, the samples were gold-plated (Quorum Q150R ES, Quorum Technologies, UK) and imaged with SEM (Tescan MIRA3 XMU, Brno, Czech Republic) in order to obtain surface images of the materials exhibiting a ‘marked change’ after medication administrations. The entire surface of the samples was scanned, and the most representative areas displaying structural surface changes were photographed at x500, x1,000, x2,000, and x5,000 magnifications with 10 kV accelerating voltage.

### Statistical analysis

The statistical analysis of the color change values was evaluated using SPSS (Statistical Package for Social Science, IBM) statistics software version 22.0. In the evaluation of the data one-way analysis of variance (ANOVA) was performed to compare the measurements, and the Tukey test was used. Paired sample t test and one-way repeated-measures analysis of variance (ANOVA) were performed to compare the measured values obtained from the same samples at different intervals, and the Bonferroni test was used. *p*-values equal to or less than 0.05 were considered statistically significant.

## Results

### Comparison of ΔEs of DRMs

**Fig. 2:**
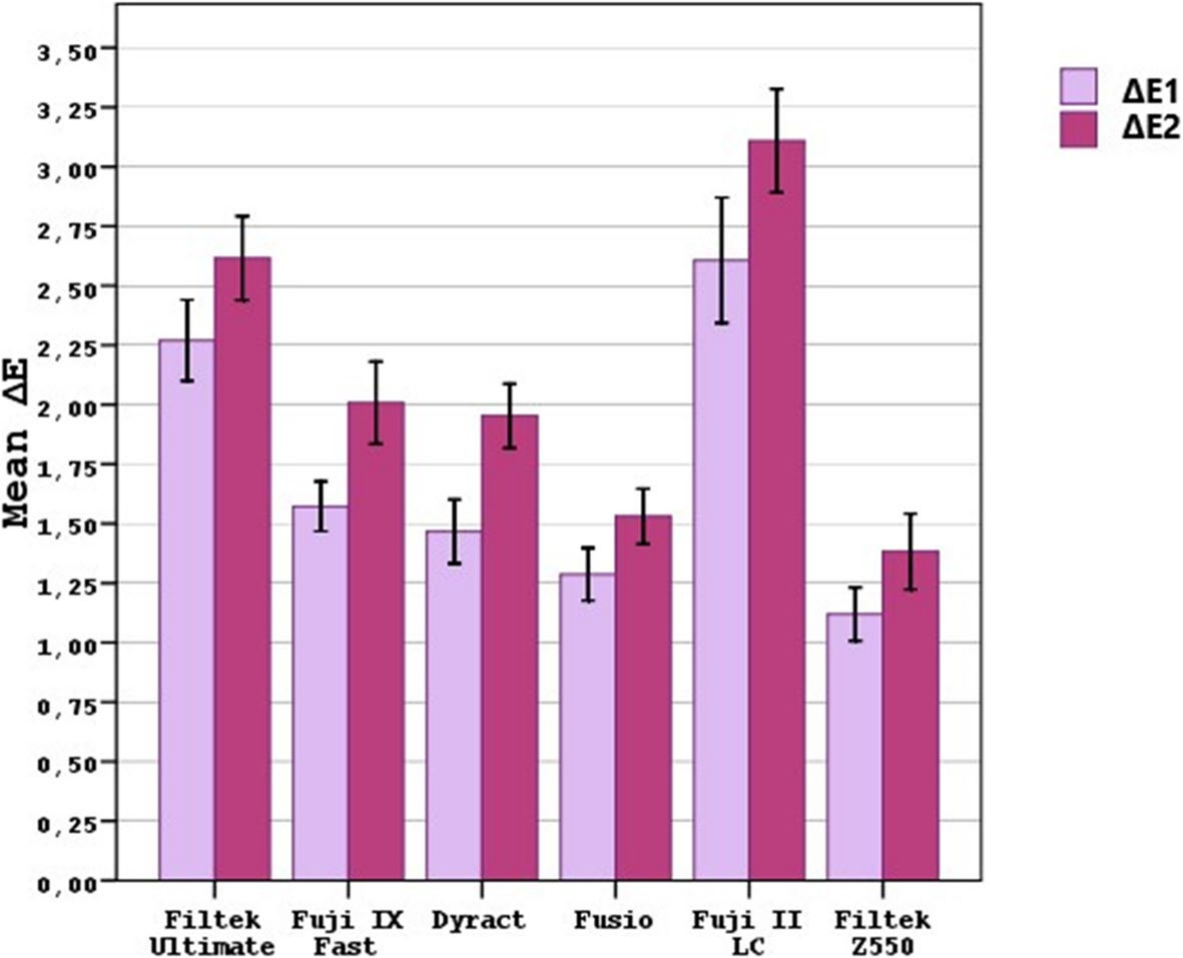
General comparison of color change values (ΔEs) of dental restorative materials

The ΔEs of samples after the administration of medications are shown in Table 3. It was observed that resin-modified glass ionomer (RMGIC) had the highest ΔE and nanohybrid composite had the lowest ΔE (Fig. 2) when comparing the difference between ΔE1 (initial − 7th days) and ΔE2 (initial – 21st days) of DRMs.

**Table 3:** The color change values (ΔE) of DRMs by IAMs and over time

		**Filtek Ultimate**	**Fuji IX Fast**	**Dyract XP**	**Fusio Likit Dentin**	**Fuji II LC**	**Filtek Z550**	**Results**
**Budecort**	**Δ1**	2,00 ± 0,20^A,a^	1,48 ± 0,21^A,b^	1,31 ± 0,27^A,b,c^	1,14 ± 0,24^A,c,d^	2,06 ± 0,20^A,a^	0,90 ± 0,08^A,d^	F = 38,65 *p* = 0,001*
	**Δ2**	2,19 ± 0,30^A,a^	1,80 ± 0,22^B,a,c^	1,70 ± 0,08^B,a,c^	1,43 ± 0,36^A,b,c,d^	2,68 ± 0,13^B,e^	1,21 ± 0,43^A,d^	F = 27,45 *p* = 0,001*
	**Results**	t = 1,70 *p* = 0,132	t = 11,81 *p* = 0,001*	t = 5,13 *p* = 0,001*	t = 1,77 *p* = 0,120	t = 5,78 *p* = 0,001*	t = 2,18 *p* = 0,065	
**Flixotide**	**Δ1**	2,25 ± 0,47^A,a^	1,47 ± 0,28^A,b^	1,34 ± 0,13^A,b^	1,21 ± 0,48^A,b,c^	1,94 ± 0,12^A,a^	0,84 ± 0,06^A,c^	F = 21,99 *p* = 0,001*
	**Δ2**	2,66 ± 0,31^B,a^	2,02 ± 0,46^B,b^	1,88 ± 0,17^B,b^	1,30 ± 0,32^A,c^	2,74 ± 0,46^B,a^	1,09 ± 0,29^B,c^	F = 29,01 *p* = 0,001*
	**Results**	t = 3,08 *p* = 0,018*	t = 8,28 *p* = 0,001*	t = 6,27 *p* = 0,001*	t = 1,19 *p* = 0,272	t = 5,23 *p* = 0,001*	t = 3,00 *p* = 0,020*	
**Ventoline**	**Δ1**	2,34 ± 0,61^A,a,b^	1,61 ± 0,44^A,a,c^	1,49 ± 0,61^A,c,d^	1,35 ± 0,23^A,c,d^	2,94 ± 0,68^A,b^	1,30 ± 0,35^A,c,d^	F = 12,93 *p* = 0,001*
	**Δ2**	2,66 ± 0,52^B,a,b^	2,07 ± 0,80^B,a,c^	1,92 ± 0,57^B,c,d^	1,56 ± 0,05^B,c,d^	3,02 ± 0,32^A,b^	1,44 ± 0,15^A,c,d^	F = 13,33 *p* = 0,001*
	**Results**	t = 4,80 *p* = 0,002*	t = 3,34 *p* = 0,012*	t = 6,67 *p* = 0,001*	t = 3,17 *p* = 0,016*	t = 0,54 *p* = 0,603	t = 1,33 *p* = 0,223	
**Combined**	**Δ1**	2,47 ± 0,45^A,a^	1,70 ± 0,08^A,b^	1,71 ± 0,17^A,b^	1,44 ± 0,05^A,b^	3,46 ± 0,14^A,c^	1,42 ± 0,09^A,b^	F = 109,90 *p* = 0,001
	**Δ2**	2,94 ± 0,50^B,a^	2,12 ± 0,20^B,b^	2,21 ± 0,36^B,b^	1,82 ± 0,22^B,b^	3,97 ± 0,21^B,c^	1,77 ± 0,51^A,b^	F = 43,18 *p* = 0,001*
	**Results**	t = 9,38 *p* = 0,001*	t = 9,00 *p* = 0,001*	t = 6,76 *p* = 0,001*	t = 5,70 *p* = 0,001*	t = 4,29 *p* = 0,009*	t = 2,17 *p* = 0,066	

The different uppercase letters represent the difference in the columns, the different lowercase letters represent the difference in the lines. * *p* < 0.05 was accepted as significance level

**Fig. 3:**
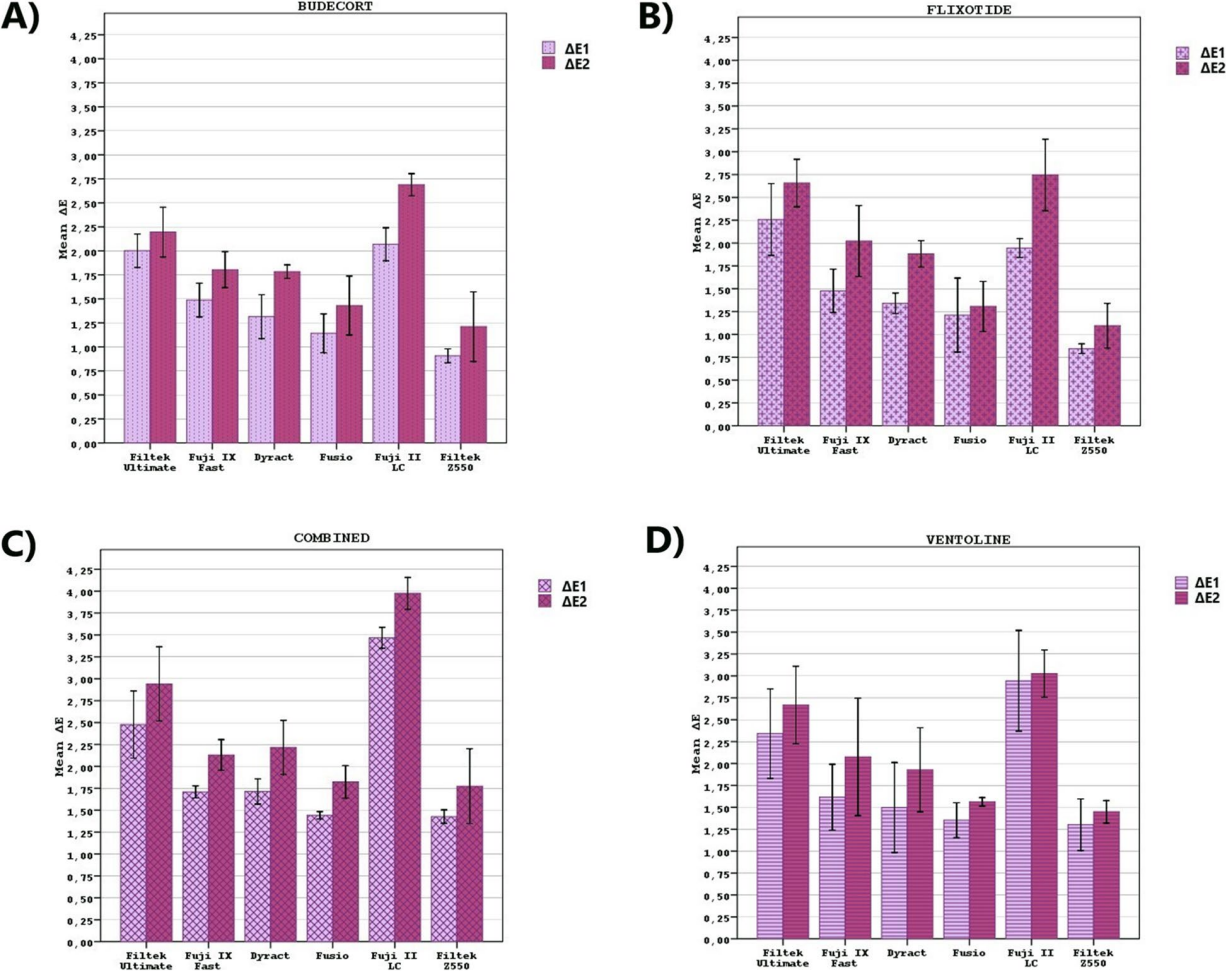
The comparison of color change values (ΔEs) of dental restorative materials at different time intervals (**A**) in the Budecort group, (**B**) in the Flixotide group, (**C**) in the combined medication group, and (**D**) in the Ventoline group

Moreover, it was determined that IAMs that most affect the ΔEs of the DRMs are Ventoline and combined medication administrations on the 7th and 21st days (Table 3; Fig. 3).

ΔE1 and ΔE2 of Budecort group were compared; the difference between ΔEs of nanofilled flowable composite, self-adhering composite and nanohybrid composite was found to be insignificant (*p* > 0.05), whereas the difference between ΔEs for other DRMs was found significant (*p* < 0.05). When ΔE1 and ΔE2 of the Flixotide group were compared; the difference between ΔEs of the self-adhering composite was found to be insignificant (*p* > 0.05), the difference between ΔEs for other DRMs was found to be significant (*p* < 0.05). ΔE1 and ΔE2 of the combined medication group were compared; the difference between ΔEs of the nanohybrid composite was found to be insignificant (*p* > 0.05) and the difference between ΔEs for other DRMs was found to be significant (*p* < 0.05). ΔE1 and ΔE2 of Ventoline group were compared; the difference between ΔEs of RMGIC and nanohybrid composite was found insignificant (*p* > 0.05), whereas the difference between ΔEs for other DRMs was found to be significant (*p* < 0.05).

### Comparison of the effects of IAMs on discoloration of DRMs

When the ΔE1s caused by the IAMs were compared on the 7th day, the difference was significant (*p* < 0.05). While the difference between combined-Budecort, Ventoline-Budecort and combined-Flixotide was significant (*p* < 0.05), the difference between other IAMs was insignificant (*p* > 0.05).

When the ΔE2s caused by the IAMs were compared on the 21st day, the difference was significant (*p* < 0.05). While the difference between combined-Budecort and combined-Flixotide was significant (*p* < 0.05), the difference between other IAMs was insignificant (*p* > 0.05).

The highest ΔEs on the 7th and 21st days were obtained in the combined and Ventoline medication groups.

### Visual results of the color change values of the DRMs

**Fig. 4:**
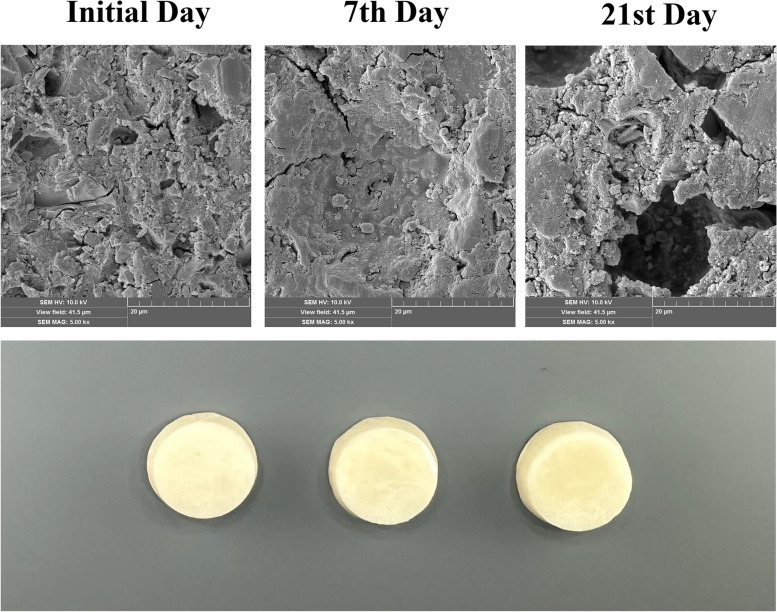
The initial, 7th, and 21st day SEM images and photographs of the combined medication-administered Fuji II LC material

DRMs were affected by inhaled medications during the experimental period, but their color change values did not exceed the ‘Appreciable: marked change’ threshold, with the exception of RMGIC in the combined medication group on days 7 and 21. Figure 4 shows SEM images of the time-dependent surface topography of the combined medication administered Fuji II LC material.

According to the NBS ratings, ‘marked change’ occurred only when combined medication administered Fuji II LC group at all time intervals. A ‘slight change’ was observed in packable GIC, compomer, self-adhering composite and nanohybrid composite group administered Budecort, Flixotide and Ventoline medications for 7 days. The combined medication administration was able to show the same effect only in nanohybrid composite and self-adhering composite groups on the 7th day. In addition, a ‘slight change’ was observed in self-adhering composite and nanohybrid composite groups administered Budecort, Flixotide and Ventoline medications for 21 days. ‘Perceivable change’ was observed in all groups other than those mentioned above (Table 4).

**Table 4:** The color changes values (NBS Units) of DRMs by IAMs and over time

**Materials**	**Medications**	**Timepoints**	**NBS ratings**		
			**0.5–1.5 Slight:** slight change	**1.5–3.0 Noticeable:** perceivable change	**3.0–6.0 Appreciable:** marked change
**Filtek Ultimate**	Budecort	7th day		1,84	
		21st day		2,01	
	Flixotide	7th day		2,07	
		21st day		2,45	
	Combined	7th day		2,27	
		21st day		2,70	
	Ventoline	7th day		2,15	
		21st day		2,45	
**Fuji IX Fast**	Budecort	7th day	1,36		
		21st day		1,66	
	Flixotide	7th day	1,35		
		21st day		1,86	
	Combined	7th day		1,56	
		21st day		1,95	
	Ventoline	7th day	1,48		
		21st day		1,90	
**Dyract XP**	Budecort	7th day	1,20		
		21st day		1,56	
	Flixotide	7th day	1,23		
		21st day		1,73	
	Combined	7th day		1,57	
		21st day		2,03	
	Ventoline	7th day	1,37		
		21st day		1,77	
**Fusio Likit Dentin**	Budecort	7th day	1,05		
		21st day	1,32		
	Flixotide	7th day	1,11		
		21st day	1,20		
	Combined	7th day	1,32		
		21st day		1,67	
	Ventoline	7th day	1,24		
		21st day	1,44		
**Fuji II LC**	Budecort	7th day		1,90	
		21st day		2,47	
	Flixotide	7th day		1,78	
		21st day		2,52	
	Combined	7th day			3,18
		21st day			3,65
	Ventoline	7th day		2,70	
		21st day		2,78	
**Filtek Z550**	Budecort	7th day	0,83		
		21st day	1,11		
	Flixotide	7th day	0,77		
		21st day	1,00		
	Combined	7th day	1,31		
		21st day		1,63	
	Ventoline	7th day	1,20		
		21st day	1,32		

NBS units were calculated according to = ΔE^∗^ × 0.92

Since there is no material with NBS rating > 6 and < 0.5, it was not included in the table

## Discussion

According to the results of the present study, since there were differences between the color changes caused by IAMs, the null hypothesis was rejected.

Especially in anterior dental restorations, it is very substantial to know the color-resistant properties of DRMs by dental professionals in order to provide the patient with functional and esthetically long-lasting restorations. The color stability of resin-based DRMs could be affected by the type of material and light polymerization strategy [13]. The material is favored for dental restorations not only because of its mechanical properties but also because of its resistance to discoloration, particularly when used in aesthetically important areas. In the present study, restorative materials with variable proportions of filler and resin matrix were favored. To standardize the color of different DRMs, the A2 shade was used for all materials. Likewise, all samples were prepared with a standard mold in order to ensure equal and maximum polymerization in all parts of the materials. The oxygen inhibition layer is formed as a result of the contact of the surfaces of resin-based DRMs with air. This may affect the polymerization quality of the outer layer of resin-based DRMs [13, 14]. In the present study, mylar strips were placed on the lower and upper surfaces of the materials and polymerized in order to minimize the layer formed in resin-based DRMs as well as to obtain smooth and homogeneous surfaces. After the DRMs were polymerized, finishing and polishing procedures were applied to all samples.

Among the asthma medications available in various forms such as tablets, syrups and inhalers, IAMs are very important in the treatment of asthma. Because higher drug concentrations can be achieved locally in the respiratory tract with IAMs. At the same time, this route of administration has a low risk of systemic side effects. Therefore, IAMs can be administered several times a day using various forms of inhalers or nebulizers. However, the duration of use of the IAMs used in asthma treatment may differ. While short-term medication use is necessary for acute exacerbation, regular and long-term prescription of medications is required in children with chronic asthma due to the chronic nature of the disease. In the present study’s in vitro conditions, we evaluated 7-day medication administration to mimic acute exacerbations treatment and 21-day medication administration to simulate chronic treatment for childhood asthma.

Numerous aging/staining protocols have been used in the literature to evaluate the discoloration of DRMs. Miotti et al. [15] showed that the partial immersion method of DRMs in staining solutions results in lower color change values than the total immersion method. Theoretically, it has been reported that the clinical situation is better simulated when DRMs are immersed in staining solutions by partial immersion. However, the form (inhaler form/aerosol) of the medications used in the current study differs from studies in the literature that employ aging protocols involving medications/liquids (syrups, beverages). In the present study, inhaled medications were administered vertically, in in-vitro conditions, only perpendicular to the upper surfaces of the samples, as in clinical practice, since we think that DRM surfaces bonded to teeth will not interact directly with inhaler medications.

The preferable assessment methods in the studies should be identical to facilitate a clearer comparison between the results of the research and those of the literature. In light of this, color change evaluation (CIE LAB) formulations, which have been reported to be utilized more frequently in the literature [8], were utilized in the present study.

DRMs used in dentistry must be resistant to environments that cause color change. The factors affecting the color sensitivity of DRMs are water absorption / solubility properties, surface reactivity and polimerization reaction of the materials [16]. Color perception is a complex concept influenced by various factors such as transparency, opacity, type, and direction of light. Spectrophotometric color analysis is more accurate and repeatable compared to visual color evaluation [17]. In order to reduce subjective evaluation errors when determining the colors of the DRMs, a spectrophotometer, a dependable instrument for color analysis, was utilized in this study.

When the DRM thickness is on average 1–2 mm, the effect of the background used during color measurement on the color of the restoration is significant [18]. Consistent ΔEs can be compared if the color measurement is done on the same background each time. When the effect of the background / environment on the accuracy of visual color matching was investigated, it was found that the color match was best on black and white backgrounds [19]. In a previous study [20], color measurements were made on both black and white backgrounds, and it was reported that these two backgrounds could simulate two different clinical situations. They stated that the black background can mimic the situation where there is no dental structure behind the DRM, while the white background can mimic the situation that one of the dental walls is still present. However, it is still unclear which background is more suitable for dental color measurement [18]. In light of these considerations, the thicknesses of each of the DRMs in the present study were standardized as 2 mm, and color measurements were made on a standard background in the northward corner of the same room by the same person at the same time of the day.

Clinical manifestations of childhood asthma can range from mild to difficult to treat to severe. As a result, the medications used to treat asthma and the dosages required for treatment are highly variable [1]. It was evident in the present study that combined medication administration caused a higher color change in DRMs compared to other IAMs administrations. This is probably due to the fact that the MADD for combined medication therapy is higher than other IAMs. Moreover, different levels of ingredients such as citric and sulfuric acid in IAMs could affect the surface topography of DRMs [10]. The high ΔEs observed in the samples in the Ventoline and combined medication administered groups can be explained by the high MADD of these medications and the strong acidity of the sulfuric acid in the Ventoline nebul compared to the citric acid found in Budecort.

Rinsing the mouth after nebulization of some inhaled medications, particularly after the use of corticosteroids, reduces the risk of candidiasis and alleviates the unpleasant taste in the mouth [1]. To simulate the recommented mouth rinsing after nebulization, samples were rinsed under running distilled water in the present study. In contrast, the samples were not brushed because only the color change caused by IAMs was investigated.

The water sorption property of resin based composite restorative materials can promote hydrolysis of silane coupling agents and the loss of chemical bonds between inorganic fillers, promoting degradation of materials [21]. Water absorption/solubility could vary greatly depending on the formulation of the resin composite restorative materials. This situation is mainly due to the presence of more hydrophilic or hydrophobic monomers in the resin matrix component. Depending on the containing monomer type, there are differences in the water absorption of the polymers, and the hydrophilicity of the monomers is TEGDMA > Bis-GMA > UDMA > Bis-EMA, respectively [22, 23]. When the overall color change comparison of the DRMs in the present study was evaluated, it was seen that the three DRMs (Filtek Z550, Fusio Liquid Dentin and Dyract XP) with the lowest color change values contain UDMA monomer (Fig. 2). In spite of the fact that microhybrid composite and RMGIC contain UDMA, which is less hydrophilic than Bis-GMA and TEDGMA, a recent study [24] revealed that these materials exhibit significant discoloration. Thus, it is believed that monomers alone cannot determine the staining property of resin based DRMs.

Inorganic filler size might influence the optical characteristics of resin composites; in other words, it has been reported that materials with smaller filler sizes can maintain long-term optical stability [25]. In contrast, findings in another study revealed that composite resins with smaller filler sizes do not necessarily show low discoloration [26]. On the other hand, the sensitivity of the materials to coloration is not only affected by the inorganic filler size of the composite resins [27]. In our previous study [10], the effects of IAMs on the surface topography of restorative materials were investigated, and the surface roughness of Filtek Z550 and Filtek Ultimate was found to be similar. Therefore, in the present study, higher ΔEs of nanofilled composites compared to nanohybrid composites may show that the color change that occurs is not solely dependent on particle size or surface roughness. Bociong et al. [23] found that the water sorption of the Filtek Ultimate (nanofilled composite) is higher than that of the nanohybrid composites. Consequently, the fact that ΔEs of Filtek Ultimate are higher than ΔEs of Filtek Z550 after IAMs administration in the present study, may be due to the high amount of water sorption. The discoloration that occurs in DRM is multifactorial, and it is not possible to blame a single factor.

Similar to the results of the present study, in a previous study [28] evaluating the color changes of nanohybrid composite and RMGIC, the ΔEs of the materials were found to be nanohybrid composite < nanofilled composite < RMGIC, respectively. Abu-bakr et al. [16] showed that compomer and RMGICs are susceptible to discoloration as a result of exposure to various solutions for extended periods of time. Parallel to this study, while the hybrid composite showed the minimum detectable color change, ΔEs of RMGIC were found to be higher than the compomer. In a clinical study [29], the color match of the materials was determined to be 81.3% for Dyract and 28.6% for Fuji II LC. This situation supports the idea that higher ΔEs in Fuji II LC groups compared to Dyract groups may also occur clinically in this study conducted under in vitro conditions.

The idea that conventional GIC and RMGIC cements are not equally sensitive to surface discoloration due to their different material composition [5] is supported by the results of this study. In a previous study [30], conventional GICs exhibited lower water absorption values than RMGICs (excluding Equia Forte Fil). The hydrophilic nature of HEMA, which is in the composition of RMGICs and has water sorption property, may also cause this situation, as well as Bis-GMA, UDMA, etc. can also play a role. In addition, some of the RMGICs have also been reported to have a significant reduction in water absorption after a month of storage in water, due to the conversion of “loosely bound” water to “tightly bound” water in materials over time [31]. This may explain why the color change rate in Fuji II LC in this study was high in the first 7 days with medication administration, and the color change did not increase at the same rate during the following 14 days.

Futhermore, the high content of organic resin causes RMGICs to have a higher sensitivity to discoloration than conventional GICs, while the higher water content and less water absorption of conventional GICs make them less susceptible to discoloration [5]. These results support the findings that the color change in self cured-packable GIC in this study is less than the color change occurring in RMGIC.

In the in vitro conditions of the present research, the type of medications, the administration time-dosage, and the type of DRMs are important factors affecting the susceptibility to discoloration. However, the discoloration observed clinically in DRMs may be different. It should be kept in mind that the medications may dilute with saliva during the nebulization of inhaled medications in the oral environment, and the DRMs can be mechanically cleaned by chewing or brushing.

As it is not feasible to mimic the intraoral conditions in their entirety, this in vitro research into the effect of inhaled asthma medications on the color change of restorative materials has some limitations. A single color (A2) was selected for all DRMs in the present research, and capsulated forms have been preferred for the purpose of standardizing GICs. It was not feasible to replicate the clearance consequences of salivation or ingestion during the inhaler medication administration. Moreover, the artificial saliva utilized in the study lacks the proteins and enzymes found in natural saliva. The MADDs for children aged 6 years and older, as determined by the manufacturer’s insert of each IAM, were assessed in order to standardize the dosage of IAMs for the present study. Nevertheless, patients administer medications for varying durations and dosages in accordance with their age. Therefore, there may be different medication-dosage clinical scenarios in daily life for a asthmatic patient. In addition, translucency and whiteness parameters, which are other factors affecting the aesthetic clinical acceptance of the material, were not evaluated in this study.

Considering that DRMs are widely used in asthma patients, it is aesthetically important to evaluate the color changes of different DRMs caused by IAMs. Since the medications used in the treatment of chronic asthma are used for a long time, it will be beneficial for dental professionals to be careful in the selection of DRMs to reduce the esthetic concerns of the patients. Due to the administration of inhaler asthma medications, ΔEs increased for all DRMs. As a result, the exposure time of the DRMs to the medications increased, and higher ΔEs were detected. While the highest ΔEs were recorded in combined and Ventoline medication administered groups, the material with the highest ΔE was RMGIC.

The current in vitro study demonstrates that inhaled asthma medications can influence the color stability of dental restorative materials. When selecting DRMs for pediatric asthma patients, it is essential to consider the IAMs that these patients utilize. In the present in vitro study, it was found that combined IAM treatment caused more discoloration of DRMs than single IAM treatment options. The current research may reference future clinical research and contribute to the literature.

## Why this paper is important to paediatric dentists

- Since asthma is the most common chronic disease in children, it is important to evaluate the effects of IAMs used in the treatment of this disease on DRMs.
- The present in vitro study suggests that similar clinical results of IAMs may occur. The color stability of paediatric DRMs can be affected by IAMs used in asthma treatment.
- In the selection of DRMs for paediatric asthma patients, the IAMs used by these patients should be considered. Combined IAM therapy causes more discoloration in DRMs compared to single IAM therapy.
- It can be advisable for dental practitioners to recommend that children who use inhaler medications adopt more vigilant oral hygiene practices and schedule regular visits to the paediatric dentist.

## Data Availability

The datasets used and/or analysed during the current study are available from the corresponding author on reasonable request.
